# Effects of Short-Chain Fatty Acid Combinations Relevant to the Healthy and Dysbiotic Gut upon *Candida albicans*

**DOI:** 10.1007/s00284-025-04400-0

**Published:** 2025-07-29

**Authors:** Emer Hickey, Ian Leaves, Arnab Pradhan, Qinxi Ma, Raif Yuecel, Neil A. R. Gow, Gordon D. Brown, Alistair J. P. Brown

**Affiliations:** 1 MRC Centre for Medical Mycology, Geoffrey Pope Building, University of Exeter, Exeter, EX4 4QD UK; 2 Exeter Centre for Cytomics, The Bioeconomy Centre, University of Exeter, Exeter, UK

## Abstract

The major fungal pathogen, *Candida albicans*, exists as a commensal in the gastrointestinal tract of healthy humans. Fungal colonisation levels increase during gut dysbiosis, when the local microbiota and short-chain fatty acid (SCFA) concentrations become perturbed. Individually, acetic, propionic and butyric acids are reported to exert differential effects on *C. albicans*. In this study, we tested whether combinations of these SCFAs, at concentrations that broadly reflect healthy and dysbiotic gut profiles, influence virulence-related phenotypes. The selected healthy and dysbiotic SCFA mixes slowed the growth of *C. albicans* SC5314, increased resistance to cell wall stresses (Calcofluor White, SDS, caspofungin), differentially affected the exposure of the key cell surface pathogen-associated molecular patterns (PAMPs) β-1,3-glucan, chitin and mannan, and influenced total chitin content compared with non-SCFA controls. However, few differences were observed between the healthy and dysbiotic mixes. Furthermore, comparison of isolates from other epidemiological clades revealed that most effects of the SCFA mixes were strain-specific, reflecting the high degree of phenotypic variation reported previously between clinical isolates. Interestingly, the healthy SCFA mix inhibited hyphal development to a greater extent than the dysbiotic mix in some *C. albicans* isolates including SC5314. This was not reflected in differential adhesion to Caco-2 cells or in altered virulence in the *Galleria* model of systemic candidiasis. We conclude that SCFA mixtures reflecting those present in the human gut subtly influence some virulence-related phenotypes in *C. albicans* in a strain-specific manner.

## Introduction

*Candida albicans *is arguably unique amongst fungal pathogens due to its ability to transition between commensal and pathogenic states, the variety and frequency of infections it causes, and the extensive research on its pathobiology [1].

*C. albicans* is frequently found in the intestinal microbiota of humans and is the *Candida* species most frequently isolated from the faeces of healthy humans [2, 3]. During intestinal colonisation, *C. albicans* interacts with multifarious microbes that influence the ability of this fungus to establish itself in this niche. The microbiota of the mouse intestine generally inhibits *C. albicans* colonisation [4, 5], and probiotic bacteria can limit the severity of *C. albicans* infections in immunocompromised mice and germ-free mice [6]. In humans, *C. albicans* can colonise the healthy gut, but the likelihood of developing candidemia is enhanced by treatments with broad-spectrum antibiotics [7].

The intestinal microbiota inhibits *C. albicans* colonisation partly by generating molecules that directly impact the fungus and/or by eliciting host responses that target the fungus [1, 8–10]. For example, *Lactobacillus rhamnosus* inhibits *C. albicans* morphogenesis by producing an exopolysaccharide and the chitinase Msp1 [11, 12]. *Enterococcus faecalis* attenuates fungal morphogenesis and virulence by secreting the peptide EntV [13]. Bacterial-derived metabolites affect *C. albicans* proliferation [14], and SCFAs in particular have been implicated in promoting gut-barrier integrity, immune regulation, anti-inflammatory responses and the excretion of antimicrobial functions [15].

Antibiotic-driven changes in the gut microbiota lead to decreased levels of bacterial-derived SCFAs [16], and increased fungal colonisation and dissemination [17–19]. Acetic, propionic and butyric acids are the most abundant SCFAs produced by microbial fermentation of indigestible polysaccharides, simple sugars, sugar alcohols and unabsorbed or undigested proteins in the intestine [15, 20]. SCFAs can act as weak acid stressors, but the colon (pH ~ 6.5) lies above the pK_a_ for acetic, propionic and butyric acid [21–23]. SCFA abundances vary between individuals, influenced by their microbiota and diet [24], and low SCFA concentrations can be used as biomarkers of a dysbiotic gut microbiome [25].

SCFAs attenuate *C. albicans* growth [26] and butyrate inhibits yeast-hypha morphogenesis [27]*.* SCFA resistance in *C. albicans* is dependent on Mig1 [28] and Hgt16 [29]. These proteins are likely to enhance glycolytic flux as Mig1 is a regulator of glucose repression [30, 31] and Hgt16 is a putative glucose transporter. This would be consistent with the demand for metabolic energy during stress adaptation [32]. Stress adaptation involves neutralisation of the stress, the repair of stress-mediated damage, plus the requisite energy generation via the upregulation of genes involved in glycolysis, ATP synthesis and mitochondrial respiration [22, 29, 33–36]. Consequently there are strong links between carbon source and stress resistance in *C. albicans* [31, 37, 38]*.*

Previous studies have tended to examine the influence of individual SCFAs upon *C. albicans* [28, 39–41]. However, the fungus is generally exposed to combinations of SCFAs in vivo, and *C. albicans* can display unexpected sensitivities to combinatorial inputs [42, 43]. Therefore, our aim was to test whether SCFA mixtures that reflect healthy or dysbiotic colons [18, 44–47] differentially affect *C. albicans* phenotypes involved in intestinal colonisation and virulence. Here, we describe the impact of such SCFA mixtures upon growth, yeast-hypha morphogenesis, the exposure of cell wall-associated pathogen-associated molecular patterns (PAMPs), adhesion and virulence.

## Materials and Methods

### Strains and Growth Conditions

The following *C. albicans* clinical isolates were used in this study: SC5314 (clade 1; bloodstream) [48]; IHEM16614 (clade 2; oropharynx), J990102 (clade 3; vagina) and AM2005/0377 (clade 4; oral commensal) [49]; and CEC3544 (clade 1; commensal), CEC3610 (clade 4; commensal), CEC3638 (clade 3; commensal), CEC3662 (clade 1; invasive) and CEC3669 (clade 2; superficial) [50].

*C. albicans* cells were grown in SD (2% glucose, 0.67% yeast nitrogen base without amino acids) or on YPD agar (2% agar, 2% glucose, 1% yeast extract, 2% Bacto Peptone). To prepare SCFA-containing media, stock solutions of acetic acid (Sigma-Aldrich), butyric acid (Sigma-Aldrich) and propionic acid (Sigma-Aldrich) were added at the specified final concentrations to SD, the media buffered to pH 6.5 using 2-(N-morpholino)ethanesulfonic acid (MES) (Fisher), and filter sterilised. For phenotypic analyses, *C. albicans* cells were grown overnight in SD at 37 °C with shaking at 200 rpm. These cells were used to inoculate fresh media at a starting OD_600_ of 0.2, grown for 3 h at 37 °C with shaking (200 rpm), and harvested for analysis.

### Stress Resistance

**Fig. 1:**
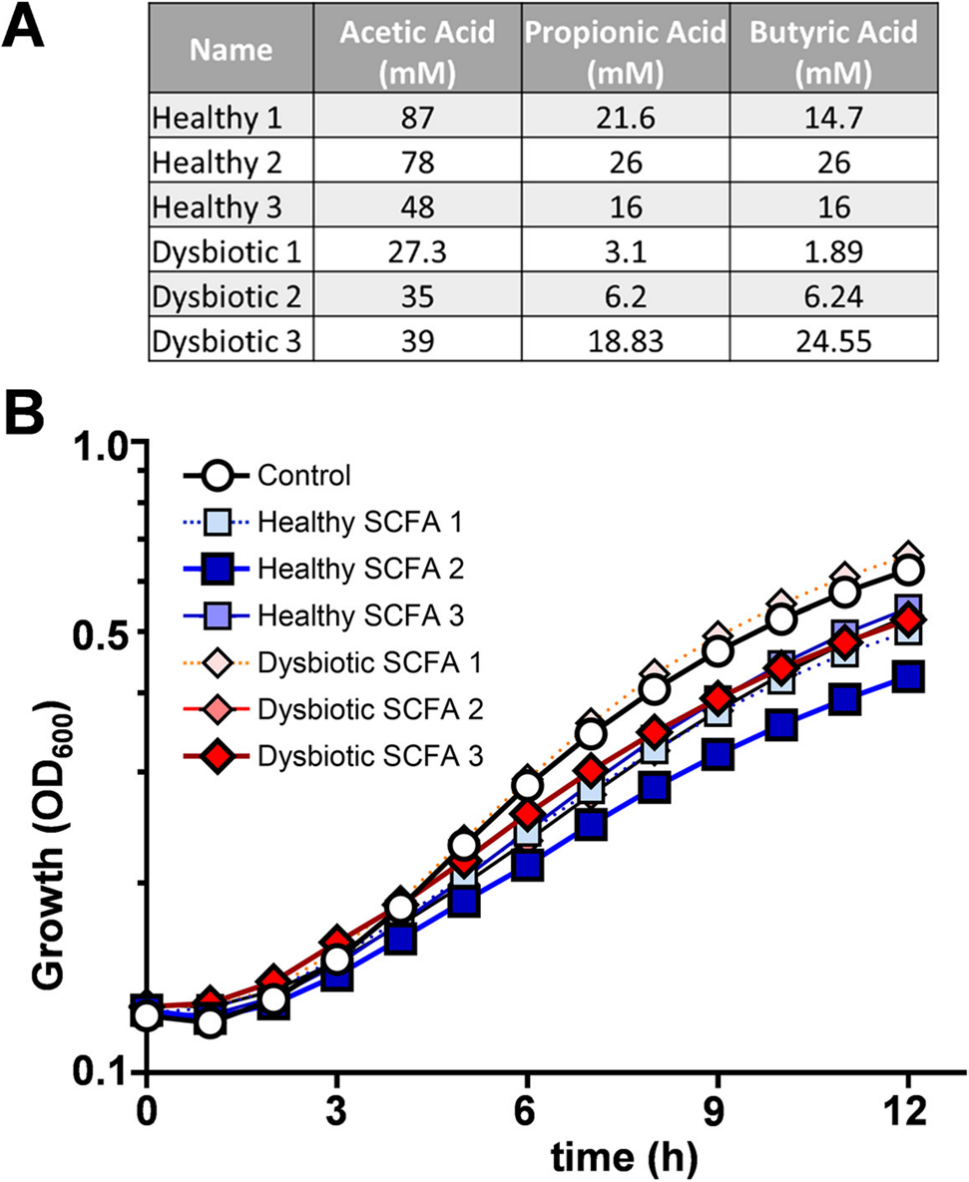
SCFA mixes impact the growth of *C. albicans* SC5314. **a** SCFA concentrations in mixes designed based on those observed in healthy and dysbiotic human guts (see text). **b** The growth of *C. albicans* SC5314 in SD containing no SCFAs (control), the three healthy SCFA mixes, or the three dysbiotic SCFA mixes (see key). The mixes selected for subsequent experiments were healthy mix 2 (dark blue squares) and dysbiotic mix 3 (red diamonds). Note that the points for dysbiotic mix 2 lie underneath those for healthy SCFA mixes 1 and 3. Means from n = 6 replicates for one representative experiment of three independent experiments are shown

Resistance to cell wall stressors was assayed in 96-well plate format in liquid media (final volume 200 μl) using *C. albicans* cells grown overnight in SD. Cells were harvested, resuspended in sterile water, and inoculated to a final OD_600_ of 0.1 into SD (no stress control), SD containing stressor, SD containing stressor plus healthy SCFA mix, or SD containing stressor plus dysbiotic SCFA mix (Fig. 1A). Stressors included: 100 μg/ml calcofluor white (CFW), 0.5% sodium dodecyl sulphate (SDS) and 3.2 μg/ml caspofungin. Plates were sealed with a breathable film, incubated at 37 °C, and growth (OD_600_) assayed at 24 h. Thermal stress was also examined by comparing growth at 30, 37 and 42 °C. The impact of the stressor was calculated by dividing the percentage of growth (OD_600_) in the presence of the stress by the growth in the absence of stress. Means and standard deviations from six independent replicates are shown. The data were analysed using ANOVA with Turkey’s multiple comparison tests: ns, p > 0.05; **, p $\le$ 0.01; ***, p $\le$ 0.001; ****, p $\le$ 0.0001.

Stress resistance was also examined using spot assays. *C. albicans* cells were grown to exponential phase in SD, diluted to an OD_600_ of 0.2 in fresh medium, a series of tenfold dilutions prepared, and these cell suspensions spotted onto SD agar containing the specified stressor. Plates were incubated for 24–72 h at 37 °C and then imaged using a GBox (SynGene). Representative results from three independent experiments are presented.

### PAMP Exposure and Chitin Content

PAMP exposure was quantified by flow cytometry using published procedures [51, 52]. *C. albicans* strains were grown overnight in SD, subcultured into fresh SD containing the specified SCFA mix and grown at 37 °C for 3 h. These exponential cells were fixed in 50 mM thimerosal (Sigma-Aldrich) [51], washed, counted using a Vi-CELL BLU Counter (Beckman Coulter), brought to a concentration of 2.5 × 10^6^ cells/ml, and stained with Fc-Dectin-1 and anti-human IgG conjugated to Alexaflour 488 (β−1,3-glucan exposure), with wheat germ agglutinin (WGA: chitin exposure) or with Concanavalin A (ConA: mannan exposure)[52–54]. Fluorescence was quantified using a BD Fortessa flow cytometer and analysed using FlowJo v10.8.1 software. Fold changes in PAMP exposure were calculated by dividing the Median Fluorescence Intensity in the presence of SCFA mix (MFI_SCFA_) by the MFI for the control condition, SD alone (MFI_CONTROL_). Data represent means and standard deviations from three biological replicates, each of which captured 10,000 events. The data were analysed using ANOVA with Turkey’s multiple comparison test: ns, p > 0.05.

The total chitin content of *C. albicans* cells grown was measured by flow cytometry, as described previously [52]. Briefly, thimerosal-fixed cells were stained with 5 μg/ml CFW for 15 min, washed, and the MFI quantified from 10,000 events using a BD Fortessa flow cytometer, as described previously [52]. Means and standard deviations from three biological replicates were analysed using ANOVA with Turkey’s multiple comparison test: ns, p > 0.05; *, p $\le$ 0.05.

### Yeast-Hypha Morphogenesis

To assay yeast-hypha morphogenesis, *C. albicans* cultures were grown overnight in SD at 37 °C with shaking (200 rpm), diluted to an OD_600_ of 0.2 in fresh SD supplemented with 3% foetal bovine serum, and incubated for 1.5 h at 37 °C with shaking (200 rpm). Cells were then fixed overnight in 50 mM thimerosal (Sigma-Aldrich) [51], washed thrice in sterile milliQ water, stained with 5 μg/ml CFW for 5 min, and washed three times with phosphate buffered saline (PBS). Cells were resuspended in 100 μl PBS containing 2 mM EDTA. The proportion of germ tubes versus ovoid yeast cells was then quantified using an Amnis Imagestream MKII Imaging Flow Cytometer (Luminex) [55]. IDEAs v6.3 software was used to gate yeast and germ tube populations based on their cell circularity and length. Means and standard deviations from three independent replicates are shown. The data were analysed using ANOVA with Turkey’s multiple comparison tests: ns, p > 0.05; ****, p $\le$ 0.0001.

### Adhesion to Caco-2 Cells

Caco-2 cells were cultured in DMEM containing 10% FCS at 37 °C with 5% CO_2_ and seeded into 24-well plates at 1 × 10^5^ cells/ml in fresh medium. After 3 days, once a confluent Caco-2 cell layer had formed, 1 ml of fresh pre-warmed medium was added. Meanwhile, exponential *C. albicans* cells, grown in SD containing or lacking SCFAs, were harvested, washed, resuspended in PBS, counted, and adjusted to 2 × 10^5^ cells/ml in pre-warmed DMEM without FBS. Yeast suspensions were added to the 24-well plate (500 μL per well) and incubated with the Caco-2 cells at 37 °C with 5% CO_2_ for 1 h. Medium and non-adhering fungal cells were then removed and remaining plated onto YPD agar to quantify adherent fungal cells (CFUs). Data represent means and standard deviations from two independent replicate experiments, each with 12 technical replicates. The data were analysed ANOVA using Turkey’s multiple comparison tests: ns, p > 0.05.

### Virulence Assays

*C. albicans* virulence was assayed in the *Galleria mellonella* model of systemic candidiasis, as described previously [53]. *C. albicans* cells were grown overnight in SD at 37 °C, subcultured into SD with or without an SCFA mix and harvested in exponential phase, as described above. The cells resuspended in sterile PBS at 1 × 10^7^ cells/ml. These cell suspensions (10 μl) were injected through the last proleg of *G. mellonella* larvae (n = 20 larvae per condition), the larvae incubated at 37 °C, and their survival monitored daily. Survival curves were compared using the Log-Rank (Mantel-Cox) test and the Logrank test for trends.

## Results

### Effects of Healthy and Dysbiotic SCFA Mixes upon *C. albicans* Growth

Our overall aim was to test whether SCFA mixtures encountered in the healthy and dysbiotic gut affect commensal- and virulence-associated phenotypes in *C. albicans*. SCFA abundances can vary between individuals and are impacted by many factors including, but not limited to, diet, microbiome composition and host health [56, 57]. Therefore our first objective was to select SCFA mixtures based on previously published literature that broadly reflect those reported for ‘healthy’ and ‘dysbiotic’ human gut profiles [18, 44–47]. We focussed on the most abundant SCFAs (acetic, propionic and butyric acids).

Initially we designed three SCFA mixtures reflecting the healthy state, and three for the dysbiotic state (Fig. 1A). Healthy SCFA concentrations were selected based on studies assessing SCFA concentrations in human cohorts [44, 45, 58]. The dysbiotic SCFA concentrations we selected from studies looking at the impact of antibiotics and disease (in this case colorectal cancer) on SCFA concentrations in rodents and humans [46, 47]. Where SCFA concentrations were obtained from rodent studies, we applied the percentage changes in SCFAs induced by antibiotics in mice to the healthy SCFA mix 2 to generate humanised dysbiotic SCFA concentrations (Fig. 1A). The mixes were designed to reflect typical healthy or dysbiotic SCFA concentrations rather than the full range of concentrations observed. We did not include single SCFA controls in our assays as the effects of individual SCFAs have been published [22, 37, 59–62].

We tested whether exposure to any of the six SCFA mixes influences growth of the commonly used clinical isolate, *C. albicans* SC5314, in minimal medium. Most of the SCFA mixes, except for dysbiotic mix 1, slowed the growth of the fungus when compared to the control lacking SCFAs (Fig. 1B). Dysbiotic mix 3 was selected for subsequent experiments as it reflects SCFA concentrations observed in a diseased human cohort (colorectal cancer patients). Healthy SCFA mix 2 was chosen because, of all the mixes examined, it inhibited *C. albicans* growth to the greatest extent.

### Impact of SCFA Mixes upon *C. albicans* Cell Wall Stress Resistance

**Fig. 2:**
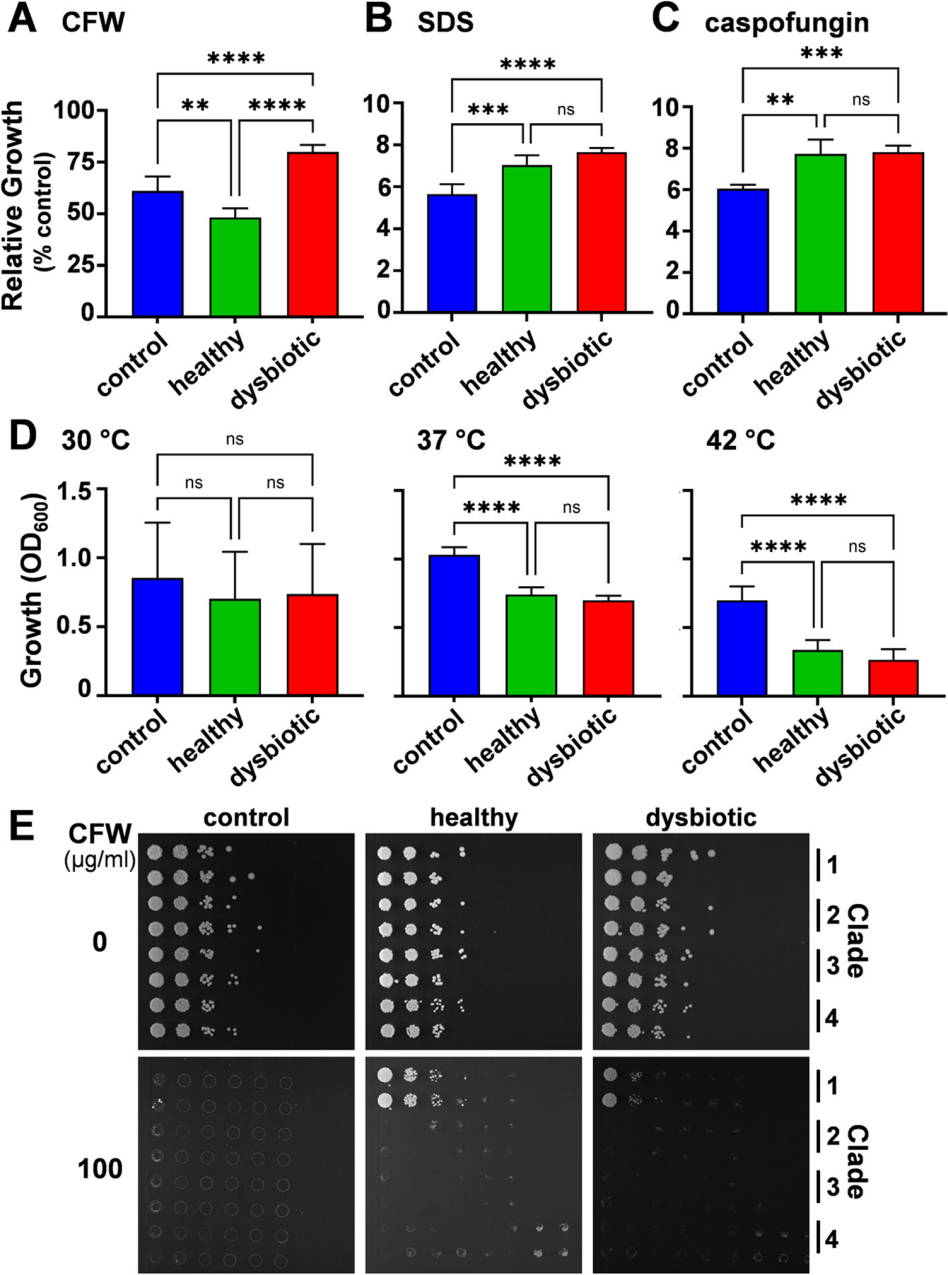
The impact of SCFA mixes on *C. albicans* cell wall stress phenotypes. *C. albicans* SC5314 cells were grown at 37 °C in SD without SCFAs (blue), with healthy SCFA mix 2 (green), or with dysbiotic SCFA mix 3 (red) in the presence or absence of cell wall stress: **a** 100 μg/ml CFW; (**b**) 0.5% SDS; or (**c**) 3.2 μg/ml caspofungin. Growth after 24 h in the presence of stress was measured (OD_600_) as a percentage of growth in the absence of stress. **d** Thermal sensitivity was determined by measuring the growth after 24 h of *C. albicans* SC5314 cells in SD at 30 °C, 37 °C, or 42 °C. Means and standard deviations from triplicate experiments are shown. The data were analysed using ANOVA with Turkey’s multiple comparison tests: ns, not significant; *, p < 0.05; **, p < 0.01; ***, p < 0.001; ****, p < 0.0001. **e** The CFW resistance of *C. albicans* isolates from different clades was compared: clade 1, SC5314; clade 2, IHEM16614; clade 3, J990102; clade 4, AM2005/0377. Duplicate series of dilutions of each isolate were plated onto SD agar, supplemented with glucose, containing no SCFAs, healthy SCFA mix 2, or with dysbiotic SCFA mix 3, and 0 or 100 μg/ml CFW, and imaged after 48 h

To determine whether these SCFA mixes influence sensitivity to cell wall stresses, phenotyping assays were performed in 96-well plate format. *C. albicans* SC5314 cells were inoculated into SD containing healthy SCFA mix 2, dysbiotic SCFA mix 3, or no SCFAs. Various cell wall stressors were added [100 μg/ml Calcofluor White (CFW), 0.5% sodium dodecyl sulphate (SDS), or 3.2 μg/ml caspofungin], and growth at 37 °C quantified by monitoring the OD_600_ after 24 h. Growth in the presence of SCFA mixes was also compared at 30, 37 and 42 °C because thermal stress is known to affect the cell wall [63, 64]. The presence of SCFAs significantly influenced the resistance of *C. albicans* SC5314 cells to each of the cell wall stresses examined (Fig. 2A–D). The SCFA mixes increased resistance to SDS and caspofungin, but reduced resistance to thermal stress (Fig. 2B–D). However, no significant differences were observed between the healthy and dysbiotic SCFA mixes in terms of their effects upon resistance to these stresses. Interestingly, under these experimental conditions, *C. albicans* SC5314 was more sensitive to CFW in the presence of the healthy mix, and more resistant to CFW with the dysbiotic SCFA mix (Fig. 2A).

Given the potential significance of elevated cell wall stress resistance in vivo, we tested whether the differential effects of the SCFA mixes upon CFW resistance represented a general or strain-specific phenomenon. To achieve this, we compared the CFW resistance of *C. albicans* isolates from different epidemiological clades on agar plates containing the different SCFA mixes. Under these conditions (growth on plates rather than in broth), *C. albicans* SC5314 cells displayed elevated CFW resistance with both healthy and dysbiotic SCFA mixes (Fig. 2E), as opposed to CFW sensitivity with the healthy mix (Fig. 2A). Differences in local pH, aeration and/or quorum sensing may account for variations in stress sensitivities observed between microtiter well and plate assays [22, 65]. Significantly, the isolates from clades 2–4 did not display elevated CFW resistance in response to either SCFA mix (Fig. 2E). We conclude that SCFA-induced CFW resistance is a strain-specific phenotype, rather than a general phenomenon in *C. albicans*.

### Healthy and Dysbiotic SCFA Mixes Do Not Differentially Affect Cell Wall PAMPs or Chitin Content

Exposure to certain individual SCFAs has been shown to influence the exposure of the proinflammatory PAMP β−1,3-glucan at the *C. albicans* cell surface, and this affects the ability of innate immune cells to recognise the fungus and trigger antifungal immune responses [61]. Butyrate enhances β−1,3-glucan exposure [52], whereas lactate masks this PAMP by inducing the shaving of exposed β−1,3-glucan from the cell surface [61, 66]. These studies examined the impact of individual SCFAs on β−1,3-glucan exposure. Here, we examined the combinatorial effects of healthy and dysbiotic SCFA mixes upon the exposure of additional cell surface PAMPs.

**Fig. 3:**
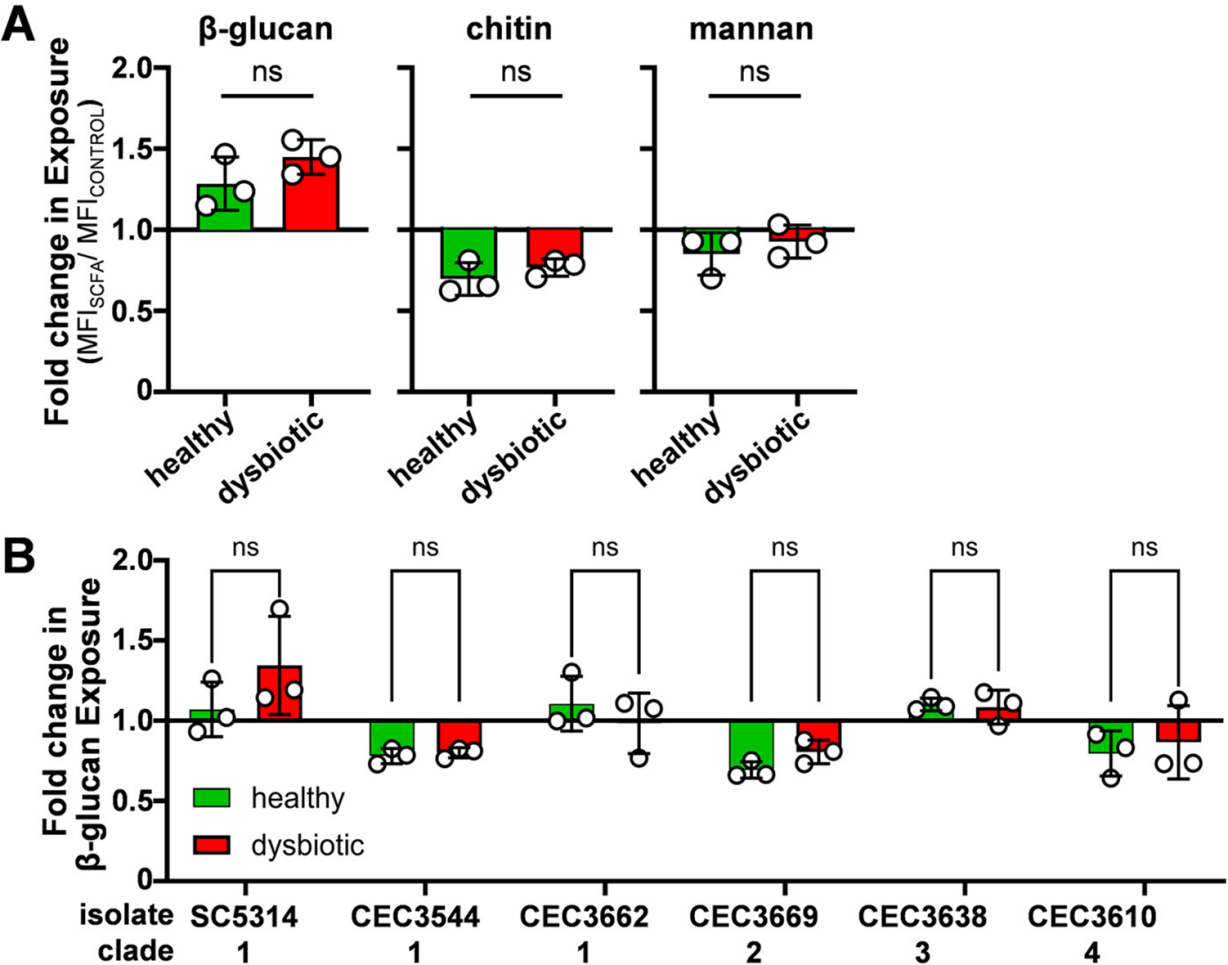
Influence of SCFA mixes on PAMP exposure at the *C. albicans* cell surface. **a** *C. albicans* SC5314 cells were grown in SD containing the healthy or dysbiotic SCFA mix and their fold changes in β−1,3-glucan, chitin and mannan exposure measured relative to control cells grown without SCFAs. Fixed cells were stained with Fc-dectin-1 (β-glucan), wheat germ agglutinin (chitin) and Concanavalin A (mannan) and their fluorescence quantified by flow cytometry. To calculate fold changes in exposure, an MFI for SCFA-treated cells was divided by the corresponding MFI for untreated cells. **b** The effects of the SCFA mixes upon β−1,3-glucan exposure were compared for *C. albicans* clinical isolates from different clades. Means and standard deviations from three independent replicates are shown, and the data were analysed using ANOVA with Turkey’s multiple comparison test. No statistically significant differences between healthy and dysbiotic SCFA samples were observed

To determine the impact of the SCFA mixes upon β−1,3-glucan, chitin and mannan exposure at the *C. albicans* cell surface, SC5314 cells were harvested during exponential growth on SD containing or lacking the SCFA mixes. These cells were harvested, fixed, stained with Fc-Dectin-1 (exposed β−1,3-glucan), wheat germ agglutinin (WGA: exposed chitin) and Concanavalin A (ConA: exposed mannan), and the fluorescence of each quantified by flow cytometry. The impact of each SCFA mix upon each PAMP was quantified by dividing the Median Fluorescence Index (MFI) in the presence of the SCFA mix relative to the MFI in the absence of SCFA (fold change = MFI_SCFA_/MFI_CONTROL_). *C. albicans* SC5314 displayed some changes in β−1,3-glucan, chitin and mannan exposure in response to the SCFA mixes, but no significant differences were observed between the healthy and dysbiotic SCFA mixes (Fig. 3A).

Given that we had observed strain-specific differences in CFW resistance (Fig. 2E), we compared the effects of the SCFA mixes upon β−1,3-glucan exposure for *C. albicans* isolates from different clades. Differential responses were observed between strains, some displaying reductions in β−1,3-glucan exposure (e.g. CEC3544, CEC3669, CEC3610) and others showing minimal changes (CEC3662, CEC3638) (Fig. 3B). However, none of the isolates tested displayed significantly different responses to the healthy and dysbiotic SCFA mixes.

**Fig. 4:**
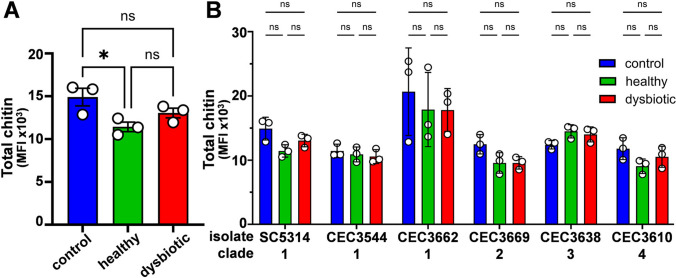
The impact of SCFA mixes on the chitin content of *C. albicans* cells. **a** The chitin content of *C. albicans* SC5314 cells was measured during exponential growth on SD (control) or SD containing the healthy or dysbiotic SCFA mix. Chitin content was assayed by staining fixed cells with CFW and measuring their fluorescence by flow cytometry. **b** The effects of the healthy and dysbiotic SCFA mixes on chitin content were compared for *C. albicans* clinical isolates from different clades. Means and standard deviations for three independent experiments are shown. The data were analysed using ANOVA with Turkey’s multiple comparison tests: ns, not significant; *, p < 0.05

Cell wall stresses, and echinocandins in particular, induce cell wall remodelling in *C. albicans*, leading to an increase in chitin content [67–69]. Therefore, we assayed the total chitin content of *C. albicans* SC5314 cells grown in the presence of the healthy or dysbiotic SCFA mixes by staining cells with CFW and quantifying their fluorescence intensity (MFI) by flow cytometry [52]. The chitin content of cells grown with an SCFA mix was found to be lower than the SCFA-free control, but only those cells grown with the healthy SCFA mix displayed a significantly reduced chitin content (Fig. 4A).

Given the strain variation we had observed for other phenotypes (above), we examined additional *C. albicans* isolates. In general, the chitin contents of six isolates from clades 1–4 decreased during growth with the healthy and dysbiotic SCFA mixes, but none of these changes were statistically significant (Fig. 4B). We conclude that, under the conditions we used, the healthy and dysbiotic SCFA mixes we examined do not differentially affect the degree of exposure of cell wall PAMPs or the total chitin content of *C. albicans* cells.

### The SCFA Mixes Inhibit Yeast-Hypha Morphogenesis

**Fig. 5:**
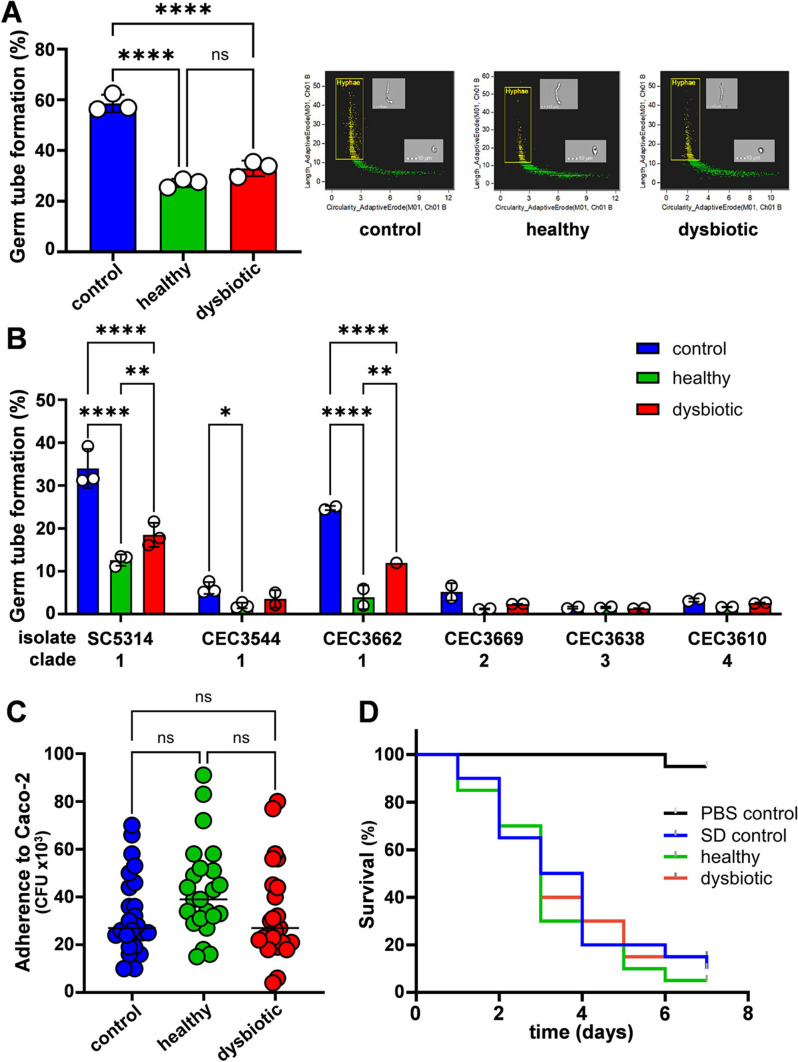
The impact of the SCFA mixes on *C. albicans* morphogenesis, adhesion and virulence. **a** Germ tube formation was measured by imaging cytometry during growth at 37 °C in SD containing foetal bovine serum. C. albicans SC5314 cells were grown with no SCFAs (control) or with healthy or dysbiotic SCFA mixes, fixed and stained with CFW to permit efficient gating and quantification of yeast cells and germ tubes. Means and standard deviations from three independent replicates are shown in the left panel. The right panels show the cytometric gating for one of these experiments. Images of gated germ tubes (hyphae) and non-gated budding cells are superimposed on histograms of the imaging cytometry outputs from samples exposed to no (control), healthy or dysbiotic SCFA mixes. **b** The impact of the SCFA mixes on germ tube formation was compared in C. albicans clinical isolates from different clades. The data represent means and standard deviations from three independent replicates and were analysed using ANOVA with Turkey’s multiple comparison tests: ns, not significant; *, p < 0.05; **, p < 0.01; ***, p < 0.001; ****, p $\le$ 0.0001. **c** C. albicans SC5314 cells were harvested during exponential growth in SD (control) or SD containing the healthy or dysbiotic SCFA mix and incubated with Caco-2 cells for 1 h, and then adherent fungal cells quantified by plating (*n* = 24 replicates). The data were analysed ANOVA using Turkey’s multiple comparison tests: ns, not significant. **d **C. albicans SC5314 cells were grown in SD (control) or SD containing an SCFA mix, 10^6^ cells or the PBS carrier alone were injected per G. mellonella larva (*n* = 20 per group), and survival of the larvae was monitored daily. No significant differences were observed between the survival curves for the SD control or SCFA mixes using the Log-Rank (Mantel-Cox) test and the Logrank test for trend

Exposure to butyrate at concentrations above 25 mM has been shown to inhibit hyphal development, whereas acetate or propionate were not inhibitory at 100 mM [27]. The healthy and dysbiotic SCFA mixes we examined contained butyrate at concentrations around 25 mM (see healthy mix 2 and dysbiotic mix 3; Fig. 1A). Therefore, we tested whether the presence of acetate and propionate in these mixes compromises the inhibitory effects of butyrate on yeast-hypha morphogenesis. To assay germ tube formation, *C. albicans* SC5314 cells were inoculated into SD containing foetal bovine serum, incubated at 37 °C for 1.5 h, and the proportion of yeast cells and germ tubes quantified by imaging flow cytometry. Both the healthy and dysbiotic SCFA mixes significantly inhibited germ tube formation (Fig. 5A). Interestingly, the dysbiotic SCFA mix appeared slightly less inhibitory than the healthy SCFA mix, but in this experiment this difference was not statistically significant.

*C. albicans* isolates display significant genetic and phenotypic variability with respect to virulence-related phenomena [1, 70–72]. Therefore, we tested whether this morphogenesis phenotype was strain-specific by examining *C. albicans* isolates from different epidemiological clades. Not surprisingly [1, 70–72], these isolates displayed differing efficiencies of germ tube formation in the absence of the SCFA mixes (Fig. 5B). CEC3638 and CEC3610 showed minimal germ tube formation under the experimental conditions analysed. In all the remaining isolates, hyphal development was inhibited by the healthy and dysbiotic SCFA mixes, and this trend was statistically significant for those isolates displaying more efficient germ tube formation (Fig. 5B). Interestingly, the dysbiotic SCFA mix was significantly less inhibitory than the healthy SCFA mix for those strains showing more than 10% germ tube formation under control conditions at the 1.5 h timepoint examined (SC5314 and CEC3662). The inference is that *C. albicans* hyphal development might be inhibited more effectively by the SCFAs in the healthy gut.

### The SCFA Mixes Do Not Affect Adhesion or Virulence

We then tested whether the SCFA mixes affect the ability of *C. albicans* to adhere to Caco-2 cells, an epithelial cell line derived from human colon. *C. albicans* SC5314 cells were grown in SD containing the healthy or dysbiotic SCFA mix or without SCFAs, harvested, washed and incubated with Caco-2 cells for 1 h. Non-adherent fungal cells were washed from the Caco-2 monolayers, and the adherent fungal cells quantified by plating onto YPD agar (CFUs). No significant differences in adherence were observed between SCFA-exposed and non-exposed *C. albicans* cells (Fig. 5C).

The impact of the healthy and dysbiotic SCFA mixes upon *C. albicans* virulence was tested in *Galleria mellonella* larvae. This invertebrate model of systemic candidiasis has been reported to reflect fungal virulence in murine models with reasonable accuracy [73, 74]. The larvae displayed similar survival rates when injected with *C. albicans* SC5314 cells grown without SCFAs or with the SCFA mixes (Fig. 5D). We conclude that exposure to the healthy and dysbiotic SCFA mixes examined does not affect the adhesion of *C. albicans* to human colon epithelial cells or virulence in the *Galleria* infection model.

## Discussion

Our expectation was that SCFA mixtures reflecting the healthy gut might attenuate virulence-related phenotypes in *C. albicans* to a greater extent than those representing the dysbiotic gut. This expectation seemed to be borne out by the inhibitory effects of the SCFA mixes upon yeast-hypha morphogenesis. Germ tube formation was inhibited to a greater extent by the healthy SCFA mix, albeit in an isolate-dependent manner (Fig. 5). This was consistent with the earlier observations that butyrate inhibits morphogenesis [27] and that antibiotic-induced decreases in gut SCFAs promote *C. albicans* colonisation [18].

The strain-dependent influence of SCFAs upon yeast-hypha morphogenesis was interesting in the context of commensalism and virulence. Differences in hyphal development between isolates drive differential degrees of tissue penetration, damage and inflammation in the host, thereby affecting the ability of *C. albicans* to colonise mucosal tissue [75–78]. In the gut, hyphal development reduces colonisation in the absence of the local microbiota [79–81], but enhances colonisation in the presence of the microbiota [82]. Hence, hyphal development lies at the heart of the fungus-host-microbiota interactions that mediate the delicate balance between *C. albicans* commensalism and pathogenicity [78]. Significantly, the ability of *C. albicans* isolates to colonise the mammalian gut seems to correlate with the degree to which SCFAs inhibit morphogenesis in these isolates [83]. Therefore, bacterial-derived SCFAs may reduce the competitiveness of *C. albicans* in the healthy gut in an isolate-dependent manner. Given that supplementation of drinking water with acetic and butyric acids reduced gastrointestinal colonisation by *C. albicans* in antibiotic-treated mice [84], and ignoring palatability issues, dietary supplementation with SCFAs could conceivably have therapeutic value under certain circumstances.

*C. albicans* isolates also display variability in their stress resistance and PAMP masking [50, 85]. These phenotypes influence immune recognition and virulence [86–88]. Therefore, any differential impacts between healthy and dysbiotic SCFA mixes upon these phenotypes would have been relevant in vivo. However, whilst these mixes influenced cell wall stress resistance (Fig. 2) and PAMP exposure to a limited extent (Fig. 3), no differential effects between the SCFA mixes were observed. Furthermore, the SCFA mixes did not significantly influence chitin content (Fig. 4), or adhesion to epithelial cells (Fig. 5A) under the conditions examined. Not surprisingly, no effects upon virulence were observed, even though the *G. mellonella* model has proven useful when assessing the virulence of *C. albicans* mutants with filamentation defects [73, 89, 90].

It should be noted that with a view to parsing apart the influence of these SCFA mixes from confounding factors, we employed well-defined growth media that differ significantly from conditions in the colon. For example, alternative carbon sources are known to influence stress resistance and virulence in *C. albicans* [37, 91]. Recent efforts to better replicate the intestinal environment in vitro include use of the SHIME model [92], gut microbiota medium [93] and organoids [94]. These models may provide an opportunity to study the effects of SCFA mixtures on *C. albicans* under conditions closer to the intestinal environment (such as during slow growth in the absence of sugars [95], in competition with the microbiota, or under hypoxic conditions). Ideally, such studies would compare multiple *C. albicans* isolates given the high degree of genetic and phenotypic variation between isolates both within and between epidemiological clades [1, 70, 71] and the ability of this fungus to evolve rapidly in response to local pressures [72, 80].
